# The adherence paradox: guideline deviations contribute to the increased 5-year survival of breast cancer patients

**DOI:** 10.1186/s12885-015-1765-0

**Published:** 2015-10-19

**Authors:** Christian O. Jacke, Ute S. Albert, Matthias Kalder

**Affiliations:** 1 Central Institute of Mental Health, Medical Faculty Mannheim/Heidelberg University, Square J5, 68159 Mannheim, Germany; 2 Department of Gynaecology and Obstetrics, Krankenhaus Nordwest, Frankfurt am Main, Germany; 3 Department of Gynaecology, Gynaecological and Obstetrics, Breast Center Regio, University of Marburg, Marburg, Germany

**Keywords:** Breast neoplasm, Inpatients, Guideline adherence, Quality indicators, Survival, Treatment outcome

## Abstract

**Background:**

In German breast cancer care, the S1-guidelines of the 1990s were substituted by national S3-guidelines in 2003. The application of guidelines became mandatory for certified breast cancer centers. The aim of the study was to assess guideline adherence according to time intervals and its impact on survival.

**Methods:**

Women with primary breast cancer treated in three rural hospitals of one German geographical district were included. A cohort study design encompassed women from 1996–97 (*N* = 389) and from 2003–04 (*N* = 488). Quality indicators were defined along inpatient therapy sequences for each time interval and distinguished as guideline-adherent and guideline-divergent medical decisions. Based on all of the quality indicators, a binary overall adherence index was defined and served as a group indicator in multivariate Cox-regression models. A corrected group analysis estimated adjusted 5-year survival curves.

**Results:**

From a total of 877 patients, 743 (85 %) and 504 (58 %) were included to assess 104 developed quality indicators and the resuming binary overall adherence index. The latter significantly increased from 13–15 % (1996–97) up to 33–35 % (2003–04). Within each time interval, no significant survival differences of guideline-adherent and -divergent treated patients were detected. Across time intervals and within the group of guideline-adherent treated patients only, survival increased but did not significantly differ between time intervals. Across time intervals and within the group of guideline-divergent treated patients only, survival increased and significantly differed between time intervals.

**Conclusions:**

Infrastructural efforts contributed to the increase of process quality of the examined certified breast cancer center. Paradoxically, a systematic impact on 5-year survival has been observed for patients treated divergently from the guideline recommendations. This is an indicator for the appropriate application of guidelines. A maximization of guideline-based decisions instead of the ubiquitous demand of guideline adherence maximization is advocated.

**Electronic supplementary material:**

The online version of this article (doi:10.1186/s12885-015-1765-0) contains supplementary material, which is available to authorized users.

## Background

Breast cancer (BC) is the most frequent female malignancy with approximately 1.65 million diagnosed women worldwide [1, 2]. Growing incidence and decreasing mortality rates are reported for developed countries. In Germany, general trends are confirmed and today, survival after BC is higher than in the 1990s [3].

### Guidelines before 2000

There are many reasons for these trends. The increasing effectiveness of therapy itself is certainly one crucial factor [4]. However, it is critical to distribute and implement published research from clinical trials into daily routine in a comprehensive manner. In the past, a small number of experts (St. Gallen consensus panel) interpreted actual results of trials and published the current state-of-the-art BC treatment [5–7]. Additionally, national [8] or European guidelines [9] provided treatment recommendations for physicians willing to improve their skills. Low acceptance and arbitrary application of these S1-guidelines were the norm, not the exception [10]. BC treatment depended mainly on experiences and knowledge of the physician. Counseling colleagues or quality circles met irregularly and the availability of expertise from other (medical) disciplines involved in the BC treatment was not institutionalized. Overall, health care professionals of different settings cooperated in a “free interplay” (e.g., liberally organized market) within a fragmented, but competitive German health care system [11].

### Guidelines after 2000

A common effort of all stakeholders intended to overcome these deficits and developed evidence-, consensus-, and outcome-based national guidelines for the early detection [10] and therapy [12] of BC. The application of these S3-guidelines was mandatory for centralized BC networks inspired by so called “hub & spoke” models [13–15]. Hubs were defined by academic institutions, and spokes refer to all of the related health care professionals. A network-wide monitoring of guideline application was assured by quality management systems, which became officially certified [13]. Multidisciplinary counseling was assured by expert panels (tumor conferences) hosted at comprehensive cancer centers. Integrated care models [16] were developed to overcome aforementioned infrastructural deficits.

### Effectiveness of guidelines

Only a few studies have focused on all (inpatient) therapy sequences, guideline adherence and its impact on outcome measures. In Germany, the studies of Woeckel et al. [17, 18] examined this topic and confirmed the general effectiveness of guideline adherence using time intervals 1992–2005. Based on these data, authors called for the maximization of guideline adherence.

This approach is straightforward and yields two critical assumptions. First, study design might be appropriate as long as the general effectiveness of S3-guidelines is of concern. However, if the appropriateness of medical decisions according to released and concurrent guidelines is of interest, the above mentioned approach is not adequate. S3-Guidelines were not released before 2003, and therefore time effects induced by different guidelines cannot be captured.

Second, the concluded guideline maximization hypothesis was based on the assumption that medical decisions adherent to the guidelines is appropriate. This assumption is true if all of the physical and mental conditions of the patient agree with clinical algorithms, ancillary conditions, and patients’ preferences. However, if one of these premises is not fulfilled, the physicians are encouraged to decide against guideline recommendations [10, 12].

### Aim of the study

The objective of the study is to exam the impact of process quality on 5-year overall survival. But in contrast to the above mentioned studies, process quality is assessed according to operating guidelines of time intervals (1996–97, 2003–04). Guideline adherence and divergence should be measured by a set of quality indicators defined along inpatient therapy sequences and related medical decisions. An overall adherence index is developed and two questions are examined: Is there a difference between guideline adherent and guideline divergent treated patients in terms of survival, first, within each time interval, and second, across time intervals? It is hypothesized that process quality increased over time. But in contrast to the cohort 1996–97, we expect an impact of process quality on survival for the cohort 2003–04. With respect to cross-period analysis, we expect higher survival of patients treated adherent to guidelines in 2003–04 and no survival differences of patients treated divergent from guidelines.

## Methods

### Incidence-based full population survey

All women with primary BC treatment in two general hospitals and one specialized academic hospital located in the district of Marburg-Biedenkopf (Hesse, Germany) were included (entry cohort). Patients were identified by surgical schedule lists and attendant histological affirmation of BC (ICD-10: C.50). Physicians recruited patients by explaining the aims of the study and obtained written informed consent. The relevant data were extracted from patient record files and stored in a clinical register [19–21]. The study was approved and conducted according to the Declaration of Helsinki and the local ethics committee of the Philipps University of Marburg (Germany).

### Sample selection for analysis

The entry cohort encompassed all treated patients (total “workload”), but not all patients of the entry cohort could be analysed by standardized quality indicators. Therefore, heterogeneous patient collectives with non-invasive tumours (pTis) and with distant metastasis or unknown metastasis status were dropped from further analysis to consider individual medical needs and the complexity of each therapy. This step defined the institutional-invasive samples. These were corrected by identifying non-resident patients to define regional-invasive samples [19–21].

### Exposure of cohorts

Cohort 1996–97 was exposed to the “free-interplay” of institutions. Primary BC treatment followed the S1-guidelines [6–9]. Cohort 2003–04 was exposed to an “integrated care” model defined by a certified BC center [13]. Primary BC treatment followed recommendations of the national S3-guidelines [10].

### Primary endpoint and follow-up

Five-year overall survival regardless of causes of death was defined as the primary endpoint. The start of the observation time was the date of surgical intervention. The verification of the vital status was assessed by the official registry office corresponding to each inpatient. Follow-up began in 10/2008 and ended in 2/2009.

### Covariates for risk adjustments

Available risk factors, prognostic and predictive factors for BC [22] were integrated into the Cox model. Regressors of the final model were: age at surgical intervention, binary nodal status, binary tumour size, binary hormone receptor status, and binary adherence index. The information on treatment location and application of chemotherapy served as strata variables.

### Quality indicators of medical decision-making

Quality indicators were defined alongside relevant inpatient treatment sequences: surgical intervention (tumor, lymph nodes) together with radio-oncological irradiation, and chemo- and hormone-therapy according to different risk categories [7, 12]. Pre-operative diagnostic sequences and other systemic interventions (e.g., HER2neu among others) were not available in 1996–97 and were excluded.

Quality indicators (QI) operationalized guideline recommendations in two categories. First, recommendations that should be respected by physicians if all other ancillary conditions are fulfilled were one category. This QI category translated to Guideline Adherent Decisions (GAD). Second, medical decisions against recommendations of the guidelines were defined by Guideline Divergent Decisions (GDD). It is important to note that GADs and GDDs are not always the opposite of each other (e.g., not disjunctive). For the definition of QIs according to the S1-guidelines (1996–97) and S3-guidelines (2003–04), short and long descriptions are provided (see Additional files 1 and 2).

### Adherence index

Developed QIs were aggregated into four indices concerning the adherence status of every therapy sequence. However, all QIs contributed to one overall binary adherence index. The aggregation of QIs was performed by the following methodology. First, each QI was assessed according to its category (GAD, GDD). Second, if all GADs were assessed as positive (e.g., adherent), BC treatment of one patient was preliminarily considered to be guideline adherent by the summarizing overall adherence index. But, if even one GAD did not catch up with guideline recommendations, the adherence index was devalued and considered to be guideline-divergent. Third, even when one GDD was administered as positive (e.g., divergent), inpatient primary BC therapy was classified as guideline-divergent by the overall adherence index. In this sense, only one disrespected quality indicator devalued all possible guideline-adherent indicators beforehand.

### Statistics

Univariate statistics describe clinical characteristics of the selected samples. The distributions of covariates between cohorts were compared by Chi-square-, Kruskall- Wallis-, Mantel-Haentszel Chi-square, Mann–Whitney U- or T-Tests. Derived *p*- values were adjusted for multiple testing by the Bonferroni-Holm method. Frequency counts described quality indicators, and Chi-Square tests adjusted for multiple testing with the Bonferroni-Holm method were applied. Multivariate survival analysis was performed by a Cox-regression model [23]. Multivariate survival curves were derived by the corrected group analysis method [24]. The significance level was defined by α = 5 %. SAS 9.3 software was used.

### Analysis strategy

Univariate results of sampling and distributions of important covariates are presented first. The number of developed quality indicators and their guideline adherence (divergence) of each therapy sequence and of the overall binary adherence index are presented. Finally, multivariate survival methods analyzed every period (e.g., cohort) separately, before cross-period/cohort comparisons without the adherence index and cross-period/-cohort comparisons conditioning on adherence status were performed.

## Results

### Sampling results

An entry cohort of 877 patients was reduced by 134 patients (15 %) due to loss to follow-up (1.9 %), non-assessable stage information (0.3 %), non-invasive tumours (6.3 %), non-assessable or distant metastases (5.2 %), or non-assessable margins of removed tumours (1.5 %). Excluded patients were randomly distributed over both cohorts (see percentages of Table 1), and no significant differences between included and excluded patients were detected (*p*-values not shown here). The exclusion of patient records left 743 (84.7 %) in the institutional-invasive samples and 504 (57.5 %) patients in the regional-invasive samples for analysis.

**Table 1:** Selection from entry cohort to samples of analysis

Sample	Cohort 1996–97		Cohort 2003–04		Total	
	*N*	%	*N*	%	*N*	%
Entry cohort	389	100,0	488	100,0	877	100,0
./. loss-to-follow up	0	0,0	17	3,5	17	1,9
./. No stage information available^a^	1	0,3	2	0,4	3	0,3
./. Stage 0^b^	22	5,7	33	6,8	55	6,3
./. Mx, M1^c^	20	5,1	26	5,3	46	5,2
./. Missings on marginal resection^d^	1	0,3	12	2,5	13	1,5
Institutional-invasive sample	345	88,7	398	81,6	743	84,7
./. Non-residents	107	27,5	132	27,0	239	27,3
Regional-invasive sample	238	61,2	266	54,5	504	57,5

Legend: ^a^refers to non-assessable stage information, ^b^excludes non tissue invasive tumors (pTis), ^c^excluded all non-assessable metastasis status or distant metastasis, ^d^patients without any information are excluded

### Descriptive statistics

Distributions of available risk, prognosis and predictive factors showed roughly balanced samples between cohorts (e.g., time intervals). Exceptions in the institutional-invasive sample refer to cohort 1996–97, which showed more invasive-ductal carcinomas (84 % vs 73 % in 2003–04), fewer G2- (48 % vs 71 %) and more G3-types (45 % vs 15 %), less R0-resection margins (85 % vs 95 %), and fewer patients from clinic C (63 % vs 80 %). A similar pattern was evident in the regional-invasive sample (see Table 2 for details).

**Table 2:** Distribution of available risk, prognostic and predictive factors in selected samples of analysis

		Institutional-invasive sample		Test	Regional invasive sample		Test
Variables	Statistic	1996–97	2003–04	*p*-value	1996–97	2003–04	*p*-value
Age at surgery	mean (SD)	60.4 (13.1)	59.8 (13.8)	n.s.	60.9 (13.3)	60.7 (14.1)	n.s.
Cancerous lymph nodes = 0	N (%)	211 (61)	266 (67)	n.s.	159 (67)	187 (70)	n.s.
Cancerous lymph nodes = 1–3	N (%)	70 (20)	84 (21)	n.s.	41 (17)	53 (20)	n.s.
Cancerous lymph nodes > 3	N (%)	64 (19)	48 (12)	n.s.	38 (16)	26 (10)	n.s.
pN-	N (%)	211 (61)	266 (67)	n.s.	159 (67)	187 (70)	n.s.
pN+	N (%)	134 (39)	132 (33)	n.s.	79 (33)	79 (30)	n.s.
pT1a (<= 0.5cm)	N (%)	25 (7)	29 (7)	n.s.	19 (8)	20 (8)	n.s.
pT1b (>0.5-1cm)	N (%)	40 (12)	70 (18)	n.s.	25 (11)	44 (17)	n.s.
pT1c (>1–2cm)	N (%)	133 (39)	155 (39)	n.s.	91 (38)	96 (36)	n.s.
pT2 (>2cm–5cm)	N (%)	112 (33)	123 (31)	n.s.	77 (32)	90 (34)	n.s.
pT3 (>5cm)	N (%)	5 (2)	8 (2)	n.s.	5 (2)	5 (2)	N/A
pT4 (incl. other symptoms)	N (%)	30 (9)	13 (3)	n.s.	21 (9)	11 (4)	n.s.
Invasiv-ductal MaCa	N (%)	290 (84)	292 (73)	0.015	200 (84)	197 (74)	n.s.
Invasiv-lobular MaCa	N (%)	29 (8)	59 (15)	n.s.	23 (10)	36 (14)	n.s.
Others	N (%)	26 (8)	59 (15)	n.s.	15 (6)	33 (12)	n.s.
Gx	N (%)	1 (0)	7 (2)	N/A	0 (0)	6 (2)	N/A
G1	N (%)	23 (7)	50 (13)	n.s.	20 (8)	36 (14)	n.s.
G2	N (%)	165 (48)	282 (71)	<0.001	108 (45)	186 (70)	<0.001
G3	N (%)	156 (45)	59 (15)	<0.001	110 (46)	38 (14)	<0.001
ER+	N (%)	253 (73)	305 (77)	n.s.	177 (74)	205 (77)	n.s.
ER-	N (%)	92 (27)	93 (23)	n.s.	61 (26)	61 (23)	n.s.
PR+	N (%)	266 (77)	285 (72)	n.s.	183 (77)	189 (71)	n.s.
PR-	N (%)	79 (23)	113 (28)	n.s.	55 (23)	77 (29)	n.s.
ERPR+	N (%)	290 (84)	318 (80)	n.s.	201 (85)	213 (80)	n.s.
ERPR-	N (%)	55 (16)	80 (20)	n.s.	37 (16)	53 (20)	n.s.
Rx	N (%)	34 (10)	3 (1)	N/A	23 (10)	2 (1)	N/A
R0	N (%)	292 (85)	378 (95)	<0.001	205 (86)	252 (95)	<0.001
R1	N (%)	17 (5)	17 (4)	n.s.	9 (4)	12 (5)	N/A
R2	N (%)	2 (1)	0 (0)	N/A	1 (0)	0 (0)	N/A
Pre-Menopause	N (%)	93 (27)	95 (24)	n.s.	60 (25)	61 (23)	n.s.
Post-Menopause	N (%)	252 (73)	303 (76)	n.s.	178 (75)	205 (77)	n.s.
Chemotherapy planned	N (%)	138 (40)	207 (60)	n.s.	158 (40)	240 (60)	n.s.
Clinic A + B	N (%)	128 (37)	79 (20)	n.s.	101 (42)	62 (23)	n.s.
Clinic C	N (%)	217 (63)	319 (80)	<0.001	137 (58)	204 (77)	<0.001

Legend: Several tests were not applicable (N/A) due to 20 % of cells with less than five cases, *p*-values adjusted for multiple testing

### Process quality indicators

In total, 104 quality indicators defined Guideline Adherent Decisions (51) and Guideline Divergent Decisions (53). Common QIs valid for both cohorts due to equal guideline recommendations related to the surgical strategy. A total number of 23 QIs referred to the sequences of breast conserving surgery and irradiation (BCS + RAD: 8 QIs) and the modified radical mastectomy (15 QIs).

The remaining QIs differed between time intervals, and cohort-specific conceptualization of QIs was required. Axilla treatment (1996–97: 2, 2003–04: 9) differed due to implementation of sentinel techniques in period 2003–04. Chemo- and hormone- therapy QIs (1996–97: 38, 2003–04: 32) differed due to distinct risk categories.

### Adherence indices

The application of defined QIs showed significant differences of guideline adherence between 1996–97 and 2003–04 (see Table 3). The relative share of guideline- adherent surgical treatments increased from 28.7 % (1996–97) to 52.8 % (2003–04) in the institutional-invasive sample (from 30.3 to 51.9 % in the regional-invasive sample). Chemotherapy adherence increased from 74.5 to 93.2 % (76.9 to 92.1 %) of treatments and hormone therapy from 70.1 to 84.4 % (68.1 to 83.8 %). Only the therapy sequence of lymph node dissection failed to exhibit a significant difference between cohorts due to the high quality level prior to infrastructural changes.

**Table 3:** Guideline-adherent treated breast cancer inpatients per therapy sequence and distribution of guideline divergences

*N* (%)	Institutional-invasive samples			Regional invasive samples		
	1996–97	2003–04	*p*-value^a^	1996–97	2003–04	*p*-value^a^
Surgical strategy incl. irradiation	99 (28.7)	210 (52.8)	<0.001	72 (30.3)	138 (51.9)	<0.001
Lymph node dissection	279 (80.9)	323 (81.2)	n.s.	188 (79.0)	209 (78.6)	n.s.
Planned chemotherapy	257 (74.5)	371 (93.2)	<0.001	183 (76.9)	245 (92.1)	<0.001
Planned hormontherapy	242 (70.1)	336 (84.4)	<0.001	162 (68.1)	223 (83.8)	<0.001
Adherence overall	46 (13.3)	140 (35.2)	<0.001	36 (15.1)	89 (33.5)	<0.001
Divergence overall	299 (86.7)	258 (64.8)		202 (84.9)	177 (66.5)	

Legend: All tests are adjusted for multiple testing, (n.s.) non-significant test results

The summarizing overall binary adherence index among all of the measured inpatient therapy sequences significantly increased from 13.3 % (1996–97) to 35.2 % (2003–04) in the institutional-invasive samples and from 15.1 to 33.5 %. In other words, a two-fold increase of process quality has been achieved and the relative share of treatments divergent from guidelines declined from 86.7 to 64.8 % (84.9 to 66.5 %).

### Multivariate 5-year survival estimates

#### Period-specific results

Furthermore, the impact of the overall binary adherence index on survival should be measured. Several steps of model selection-, check- and model-fit-procedures identified a relevant covariate set encompassing the developed adherence index. Estimates of the final Cox-regression model are shown in Table 4.

**Table 4:** Multivariate Cox-regression models applied to the adherence index and crucial risk, prognosis and predictive factors

Regressors	Beta	Standard	Test	Hazard-	95 % confidence interval	
	coefficient	error	*P*-value	ratio	Lower bound	Upper bound
*Cohort 1996–97, Institutional-invasive sample*						
Adherence index	−0.373	0.436	0.392	0.688	0.293	1.619
Age at surgery	−0.044	0.069	0.525	0.957	0.836	1.069
Nodal status (pN-, pN+)	1.049	0.263	<0.001	2.854	1.706	4.775
Tumor size (pT2, pT2-pT4)	0.121	0.244	0.620	1.129	0.700	1.821
Hormon receptor status (ERPR+, ERPR-)	0.602	0.313	0.055	1.825	0.988	3.371
Quadratic term of age at surgery	0.001	0.001	0.132	1.001	1.000	1.002
*Cohort 1996–97, Regional-invasive sample*						
Adherence index	−0.162	0.489	0.740	0.850	0.326	2.217
Age at surgery	−0.058	0.083	0.483	0.943	0.801	1.111
Nodal status (pN-, pN+)	1.184	0.336	<0.001	3.269	1.692	6.316
Tumor size (pT2, pT2-pT4)	0.015	0.307	0.961	1.015	0.556	1.854
Hormon receptor status (ERPR+, ERPR-)	0.664	0.389	0.088	1.942	0.907	4.162
Quadratic term of age at surgery	0.001	0.001	0.188	1.001	1.000	1.002
*Cohort 2003–04, Institutional-invasive sample*						
Adherence index	−0.135	0.350	0.699	0.873	0.440	1.733
Age at surgery	−0.089	0.080	0.266	0.915	0.782	1.070
Nodal status (pN-, pN+)	0.931	0.307	0.002	2.537	1.391	4.627
Tumor size (pT2, pT2-pT4)	0.615	0.302	0.042	1.850	1.023	3.344
Hormon receptor status (ERPR+, ERPR-)	1.196	0.310	<0.001	3.307	1.801	6.075
Quadratic term of age at surgery	0.001	0.001	0.173	1.001	1.000	1.002
*Cohort 2003–04, Regional-invasive sample*						
Adherence index	−0.638	0.467	0.172	0.529	0.212	1.320
Age at surgery	−0.151	0.090	0.093	0.859	0.720	1.025
Nodal status (pN-, pN+)	0.500	0.375	0.182	1.648	0.791	3.437
Tumor size (pT2, pT2-pT4)	0.512	0.371	0.168	1.668	0.806	3.450
Hormon receptor status (ERPR+, ERPR-)	1.048	0.367	0.004	2.853	1.389	5.861
Quadratic term of age at surgery	0.001	0.001	0.070	1.001	1.000	1.003

Legend: Confidence intervals (CI) with lower bounds (LB) and upper bounds (UB), planned chemotherapy (no, yes) and location of treatment (clinics A + B, C) were used as strata variables

Table 4 shows cohorts and samples across the statistical information. For cohort 1996–97, both samples (institutional- and regional-invasive) show the negative association between adherence index and survival. If a patient was treated according to the guidelines, the temporary affinity to die (hazard ratio) declined and the 5- year overall survival increased. However, this result is not significant. A systematic effect of adherence on survival is not evident. This result is consistent across cohort 2003–04 and defined samples. The related survival curves of multivariate survival estimates should be derived by the corrected group analysis (CGA) method. The results are shown in Table 5.

**Table 5:** Multivariate 5-year survival and event rates estimated by the corrected group analysis method

*Cohort*			Survival	Event	Hazard	95 % confidence interval		Test
	*Samples*	*Comparison groups*	rate	rate	ratio	Lower bound	Upper bound	*p*-value
1996–97	Institutional-invasive	Guideline divergence	76.8	23.2	1.612	0.690	3.766	n.s.
		Guideline adherence	84.5	15.5				
1996–97	Regional-invasive	Guideline divergence	79.9	20.1	1.293	0.500	3.348	n.s.
		Guideline adherence	83.4	16.2				
2003–04	Institutional-invasive	Guideline divergence	86.3	13.7	1.147	0.581	2.266	n.s.
		Guideline adherence	87.7	12.2				
2003–04	Regional-invasive	Guideline divergence	84.0	16.0	1.914	0.772	4.745	n.s.
		Guideline adherence	91.0	9.0				
Guideline-adherence	Institutional-invasive	1996–97	89.6	10.4	1.036	0.333	3.229	n.s.
		2003–04	89.9	10.1				
Guideline-adherence	Regional-invasive	1996–97	87.1	12.9	1.922	0.453	8.161	n.s.
		2003–04	92.2	7.8				
Guideline-divergence	Institutional-invasive	1996–97	76.4	23.7	1.665	1.113	2.490	0.013
		2003–04	84.6	15.4				
Guideline-divergence	Regional-invasive	1996–97	79.6	20.4	1.196	0.734	1.947	n.s.
		2003–04	82.5	17.5				

Legend: First row of each comparison yields higher hazard and lower/equal survival (effect coding)

**Fig. 1:**
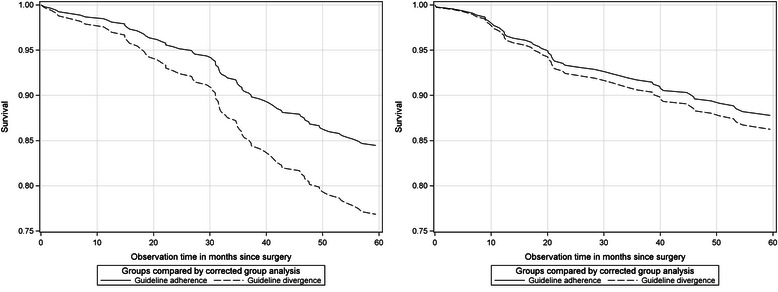
Institutional-invasive samples comparing guideline-adherent and -divergent treated patients. Cohort 1996–97 (left) and cohort 2003–04 (right)

If all of the additional variables of the Cox model are taken together, the CGA method allows for estimating survival rates and related curves [24]. The cohort- specific perspective and the institutional-invasive samples are presented first. Cohort 1996–97 exhibits remarkable survival differences between comparison groups (institutional-invasive: 84.5 − 76.8 = 7, 7). However, confidence intervals and related *p*-values indicated that the results were not significant. The same result was obtained for cohort 2003–04. A small 5-year survival difference (87.7 − 86.3 = 1,4) was estimated. However, the survival curves behave differently as Fig. 1a-b indicates.

**Fig. 2:**
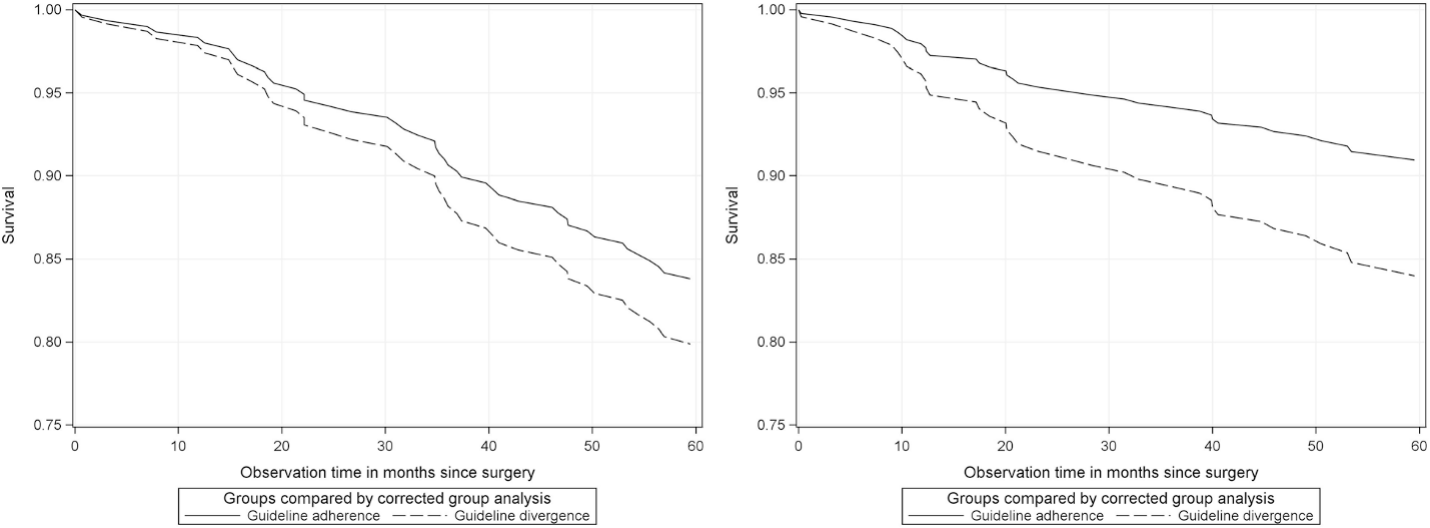
Regional-invasives samples comparing guideline-adherent and -divergent treated patients. Cohort 1996–97 (left) and cohort 2003–04 (right)

Figure 1a on the left shows the development of cohort 1996–97. The survival curves start separating after 12 months and depart after 30 months. The survival curves of guideline-divergent treated patients decline more than patients treated according to guidelines. In comparison, for cohort 2003–04 the survival differences between groups are very small, and the decline occurred after 20 months and a less steep development for the guideline-divergent treated patients was observed (Fig. 1b). If the analysis is restricted to regional-invasive samples (e.g., residential patients), cohort 1996–97 displayed small survival differences (83.4 − 79.9 = 3.5, see Table 5) and cohort 2003–04 displayed considerable survival differences (91.0 − 84.0 = 7.0, see Table 5) between the comparison groups. Figure 2a-b demonstrates insights.

Figure 2a shows the survival curve of cohort 1996–97. It seems that the curves start to separate after 12 months, and after 30 months the curve declines more. The survival curve of cohort 2003–04 (Fig. 2b) exhibits a different pattern. The survival curve starts departing from the beginning of the observation time and the curve of guideline-divergent treated patients is steeper after 10 months. Thus, the survival curves of cohorts and samples were altered substantially in terms of survival level and curve developments.

#### Cross-period results

To obtain more insights into cross-period survival rates and patterns, the cohorts were compared regardless of adherence status (not shown in tables). The institutional-invasive sample estimated a survival rate of 79 % for cohort 1996–97 and 86 % for cohort 2003–04. The survival difference between cohorts was significant (*p* = 0.007).

However, if the information of guideline adherence is added to the model and cross-period survival curves of guideline-adherent only, or guideline-divergent treated patients only were estimated, the subject becomes more intriguing.

**Fig. 3:**
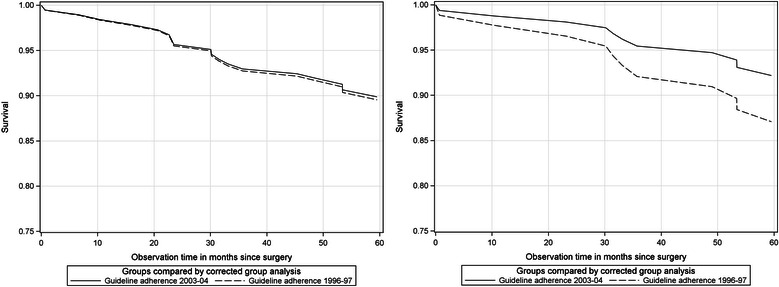
Guideline-adherent treated patients of cohort 1996–97 and cohort 2003–04. Comparison of institutional-invasive (left) and regional-invasive samples (right)

First, if only guideline-adherent patients of the institutional-invasive samples were compared, the survival estimates (see Table 5) were almost identical for cohorts 1996–97 and 2003–04 (89.6 % vs 89.9 %). The survival differences were not significant. If this comparison is restricted to residential patients (e.g., the regional-invasive sample), the survival rate of cohort 1996–97 was essentially lower than in cohort 2003–04 (87.1 % vs 92.2 %) but still not significant. Figure 3a-b shows the survival curves.

**Fig. 4:**
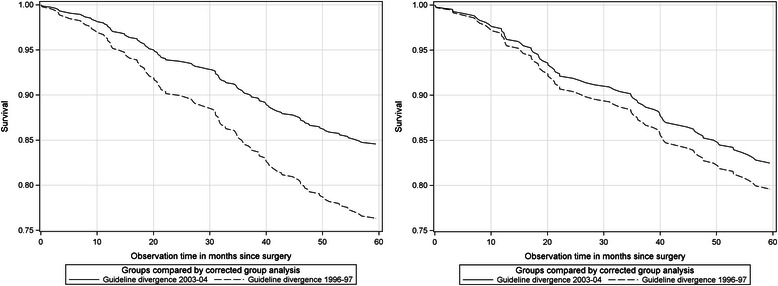
Guideline-divergent treated patients of cohort 1996–97 and cohort 2003–04. Comparison of institutional-invasive (left) and regional-invasive samples (right)

Second, only guideline-divergent treated patients were observed across the samples. The institutional-invasive samples showed a survival rate of 76.4 % in cohort 1996–97 and 84.6 % for cohort 2003–04 (see Table 5). This difference was significant (*p* = 0.013). However, this result was not replicated for the regional-invasive samples (79.6 vs 82.5; not significant). The survival curves are shown in Fig. 4a-b.

## Discussion

Based on the defined set of quality indicators according to time dependent guidelines and available medical knowledge, a two-fold increase of process quality and its medical decision making from the expert’s point of view has been observed. This result is a benefit for women with BC because the complexity of modern therapies continues to grow.

### Period-specific comparisons

The process quality of cohort 1996–97 was expected to be low, and no survival differences between comparison groups in cohort 1996–97 were expected. In fact, no impact of process quality on survival was observed. For cohort 2003–04, a higher clinical process quality was hypothesized and an impact on survival was expected. Higher survival rates of the guideline adherence group were expected but were not observed. Multivariate survival analysis revealed no significant associations of the adherence index on 5-year overall survival across all of the defined samples.

### Cross-period comparisons

The cross-period/cohort comparisons should yield deeper insights into mechanisms of temporal changes. Cross-period comparisons without considering the overall binary adherence index showed a significant difference of survival rates of approximately 7 % (see subsection ’Cross-period results’). However, cross-cohort comparisons of the adherence group only showed that estimates revealed no significant survival differences. When the guideline divergence group of cohort 1996–97 and 2003–04 were compared, systematic survival gains of 10 % were observed for the institutional-invasive sample. The latter survival increase exceeds the survival increase of periods regardless of the adherence status by approximately 3 %. This excess survival can be characterized as a period effect and was not expected for this subgroup.

In the context of guideline developments and its assessment, this unraveled period effect was deemed inconsistent with the ubiquitous demand of the maximization of guideline adherence [17, 18]. Isn’t it a paradox that particular women with BC benefited most in the last decade from treatment which violated guideline recommendations?

### Essence of guidelines

It is not inconsistent with the essence of guidelines because the identified paradox reflects the very nature of guidelines as they should apply for the vast majority of patients. Schulz et al. [10] emphasized that “if the individual situation requires deviations of guidelines, it is not solely possible, it is mandatory to do so. Guidelines do not discard physicians from their obligation to concern the clinical characteristics, somatic, psychological and social conditions of each patient”.

At this point, cohort 1996–97 and 2003–04 differ substantially from the infrastructural perspective. Systematic, rationale and conscious decisions against guidelines were made and monitored by expert panels in 2003–04.

### Why adherence paradox?

These multidisciplinary expert panels were introduced in the decade of cohort 2003–04 to cope with the essence of guidelines. Expert panels operated by leading physicians from all related disciplines (e.g., gynaecologist, oncologists, surgeons, pathologists, radio-oncologists, psycho-oncologists, etc.) gave consensual advice for further, multi-modal treatment [11]. Expert panels became an important forum to consider guideline recommendations, individual medical experience of various experts, patient preferences and their social circumstances. Expert panels use guidelines as a starting point for common recommendations and, if necessary, violate them systematically, rationally and consciously to tailor an individualized therapy. Thus, the identified adherence paradox reflects this essence of guidelines and signalizes its appropriate application in certified BC networks [15].

### Alternative approaches to define an adherence index

In comparison to related studies, most of these studies use a rate-based/criterion-based approach to define 5 to 20 quality indicators, mostly extracted from routine data [25–30]. These studies estimate that guideline adherence is between 80 and 100 %. If 33 indicators are used, the adherence of medical decisions decreases to 52 % [17, 18]. If medical decisions documented in patient record files are revised, 19 % (1993) and 54 % (1995) of 375 medical decisions appear to be adherent with current guidelines [31]. Scientifically legitimated deviations increased from 42 % (1993) to 68 % (1995). As an experimental design with the same methodology was conducted, a non-significant increase of 36 % (1996) to 40 % (1999) of 825 revised medical decisions was found [32]. Overall, the degree of adherence strongly depends on the length of observation time [33], age of the patient [34], number of quality indicators and included therapy sequences.

### Adherence index and survival of related studies

Most studies only refer to selected therapy sequences (e.g., surgery, chemotherapy, etc.) [35, 36] and dismiss effects of relevant or related interventions. Other studies assessed inpatient therapy by a small number of indicators and estimated 50 % lower hazard ratios induced by guideline adherence treatment [37]. Woeckel et al. reproduced this result with a greater number of indicators but advised that a non-linear relationship between adherence and survival seems to be persistent [17, 18]. Indeed, the influences of the socio-economic status (SES) seem to modify treatment effects because social disparities of survival have been reported [38, 39]. Hence, systematic positive and linear relationship of adherence and survival is not replicable with incomplete multivariate models. In this sense, the present study is consistent with other reports [40, 41].

### Strengths of study

Data quality assessment prior to this study [19–21] assured high data quality, epidemiological relevance, and reliable and valid survival estimates. Sample distinction between all selected patients and residential patients emphasizes that confounding effects and related biases were adjusted for survival analyses. The definition of quality indicators is based on “pathways of coherent decisions” and is superior to the rate-based/criterion-based methodology. For example, breast conserving surgery/mastectomy (BCS/MRM) together with irradiation (RAD) defines a compound therapy according to the guidelines [12]. As this approach was applied to time-interval specific guidelines, this study was able to identify the (unexpected) period effects.

### Limitations of the study

A number of the 104 quality indicators did not include important variables necessary for guideline assessment. Particularly, patients’ preferences for treatment strategies are missing. Studies have shown that up to 50 % of patients disagree with physicians’ treatment recommendations [42]. This comparatively high share of disagreement between patients (mastectomy preference) and their physicians (favoring breast conserving therapy) referring to a sample recruited between 2001 and 2003 emphasizes that guideline deviations do not descend from medical experts alone. Additionally, some indicators refer to decisions and planned actions but not to actual “clinical performance”. This limitation refers to chemo- and hormone-therapies whose time schedules strongly depend on the patients’ physical conditions. To consider this general flaw of conceptualization, new categories such as “scientifically legitimate decisions” [31, 32] or “justifiable guideline divergence” decisions [43] seem to be more appropriate to relax the rigid distinction between guideline adherence and divergence.

## Conclusions

The proof of a positive relationship of guideline adherence and survival seems to be more complex than understood so far. Unexpectedly, guideline-divergent treated patients of cohort 2003–04 benefited most. We hypothesized that infrastructural efforts made by multidisciplinary expert panels contributed to this adherence paradox. The adherence paradox reflects the essence of guidelines and signalizes appropriate application of guidelines in certified BC networks. The maximization of guideline-based decisions should substitute the postulation of adherence maximization. Finally, if women recognize treatment deviations from published patient guidelines for BC, the prognosis of therapy is no longer associated with shorter survival.

## Additional files

Additional file 1: **Common quality indicators valid across time intervals.** Quality indicators of guideline adherence and guideline divergence valid in the time-intervals 1996–97 or 2003–04. (XLSX 11 kb)
Additional file 2: **Quality indicators valid in each time-interval.** Quality indicators of guideline adherence and guideline divergence valid in each time interval 1996–97 and 2003–04. (XLSX 14 kb)
